# Trends and disparities in NIHSS reporting and outcomes in acute ischemic stroke hospitalizations: A retrospective cross-sectional study

**DOI:** 10.1007/s00701-026-06870-y

**Published:** 2026-04-21

**Authors:** Aryan Malhotra, Adam Kiss, Hao Yu, Isaac B. Thorman, Ariel Sacknovitz, Fawaz Al-Mufti, Chirag D. Gandhi

**Affiliations:** 1 New York Medical College School of Medicine, Valhalla, NY USA; 2 Department of Neurosurgery, Westchester Medical Center, Valhalla, NY USA; 3 Department of Neurology, Westchester Medical Center, Valhalla, NY USA

**Keywords:** Acute Ischemic Stroke, Disparities, Mortality, NIHSS Reporting

## Abstract

**Background:**

The National Institutes of Health Stroke Scale (NIHSS) quantifies stroke severity, yet variability in its reporting may reflect healthcare disparities and affect outcomes. This study analyzes trends in NIHSS reporting in the National Inpatient Sample (NIS) database and its relationship with patient characteristics and outcomes.

**Methods:**

This retrospective cross-sectional study used data from the NIS between May 2016 and December 2022. This nationally representative sample included patients in the US hospitalized with acute ischemic stroke (AIS). Primary outcomes include NIHSS reporting rate over time, factors associated with reporting, and subsequent discharge disposition. Propensity score matching (PSM) accounted for patient demographics, hospital characteristics, severity of illness, and use of reperfusion therapy.

**Results:**

Among 4,558,909 AIS hospitalizations, NIHSS was reported in 1,930,880 (42.4%). The proportion of hospitalizations with reported NIHSS increased from 14.33% in December 2016 to 56.27% in December 2022. After PSM, reporting of NIHSS was associated with higher odds of routine discharge (Adjusted Odds Ratio = 1.03, 95% CI: [1.01–1.04]; p < 0.001) and lower odds of inpatient mortality (0.650 [0.632–0.668], p < 0.001). Scores were less likely to be reported in females (0.970 [0.961–0.979], p < 0.001) and Black (0.963 [0.939–0.988], p = 0.004) or Hispanic patients (0.960 [0.926–0.996], p = 0.030), as well as in smaller and less urban hospitals.

**Discussion:**

Since the implementation of NIHSS reporting, rates have increased to more than half of all AIS hospitalizations as of December 2022. NIHSS reporting was significantly associated with improved discharge disposition, and patient demographics impacted odds of having a reported NIHSS, highlighting persistent disparities in stroke care.

**Supplementary Information:**

The online version contains supplementary material available at 10.1007/s00701-026-06870-y.

## Introduction

The National Institutes of Health Stroke Scale (NIHSS) is an objective tool for systematically grading stroke severity, enabling comparisons of stroke care across institutions and supporting analyses of treatment efficacy and quality improvement. Initially designed to quantify neurological recovery in tissue-plasminogen activator (tPA) therapy, the NIHSS has evolved into a critical tool for guiding stroke management and predicting outcomes [8]. Stroke severity, as defined by NIHSS scores, plays a significant role in tPA eligibility guidelines and clinical decision-making [6, 24]. Randomized controlled trials, such as the Trial of Org 10,172 in Acute Stroke Treatment (TOAST), demonstrated the prognostic power of the scale in projecting in-hospital mortality, greatly increasing its prominence as a clinical tool for guiding stroke care [1]. Further studies confirmed the predictive ability of the NIHSS across multiple healthcare centers [17, 29].

Despite its established utility, routine NIHSS scoring has only recently become widely recorded and available in administrative health databases. Certification in NIHSS scoring is also now recommended for hospitals seeking primary stroke center (PSC) designation [21, 22]. Additionally, the Centers for Medicare and Medicaid Services (CMS) have included reporting of NIHSS scores within Risk-Standardized Mortality Rate regression models as of 2022, with omission subsequently impacting performance indices [9]. At minimum, recording of presenting NIHSS score was mandated as per FY2018 *International Classification of Diseases*, *Tenth Revision*, *Clinical Modification* (ICD-10-CM) guidelines, motivating increased utilization [10].

In Q4 2016, CMS created ICD-10-CM codes for NIHSS reporting. Contemporaneously, the National Inpatient Sample (NIS) database began inclusion of scoring. Previous research has shown that in the initial national reporting period (Q4 2016), NIHSS scores were reported in only 14% of acute ischemic stroke (AIS) hospitalizations [28]. While other studies have demonstrated a marked improvement from this, reporting rates remain inconsistent; as of 2023, only 52.7% of AIS hospitalizations in Medicare fee-for-service datasets included NIHSS scores [25, 30]. Prior studies have also revealed the significant alignment between NIHSS scores and stroke mortality predictions, stressing the value of further analysis of scores to identify further trends in AIS diagnosis and recovery [28].

In this work, we performed a comprehensive assessment of NIHSS reporting rates, in-hospital mortality, and routine discharge using data from the NIS spanning Q2 2016 to FY2022. To our knowledge, our analysis provides the largest longitudinal overview of NIHSS reporting within a national public health database, focusing on trends in reporting rates over time, characterization of scoring distribution, factors associated with NIHSS reporting likelihood, and the influence of NIHSS reporting on inpatient outcomes.

## Methods

### Data source and patient selection

A retrospective cross-sectional study was conducted utilizing data from the NIS database, the largest publicly available all-payer inpatient care database in the United States. Developed by the Agency for Healthcare Research and Quality as part of the Healthcare Cost and Utilization Project (HCUP), the NIS approximates a 20% stratified sample of discharges from U.S. community hospitals, covering over 97% of the U.S. population and including approximately 7 million hospital stays annually [2]. Hospitalizations for AIS were identified using ICD-10-CM diagnostic codes (Table S1) [19]. Those involving reperfusion therapy, specifically intravascular thrombolysis (IVT) and endovascular thrombectomy (EVT), were identified using procedure codes from the *International Classification of Diseases*, *Tenth Revision*, *Procedure Coding System* (ICD-10-PCS) [34]. The NIS is publicly available and all patient data is de-identified; consequently, individual informed consent and institutional review board approval were not required for this study. This study conforms to the Strengthening the Reporting of Observational Studies in Epidemiology (STROBE) reporting guidelines for observational studies.

### Data characteristics and outcomes measured

While the ICD-10-CM codes for NIHSS scores were reportedly created in October 2016, our analysis discovered NIHSS reporting beginning in May 2016 (Table S2). Accordingly, data utilized in this study starts with May of 2016 in concordance with the beginning of NIHSS score reporting. According to the ICD-10-CM Official Guidelines for Coding and Reporting FY2024, the NIH Stroke Scale (NIHSS) codes (R29.7–) can be used in conjunction with acute stroke codes (I60-I63) to identify the patient's neurological status and the severity of stroke [11, 18, 31].

The primary outcome measured was the presence of a reported NIHSS score. The total number of patients identified with AIS and with reported NIHSS scores was tabulated and plotted by month. Secondary outcomes included routine discharge and inpatient mortality, defined by values of “1” and “20,” respectively, under the HCUP data element “DISPUNIFORM.”

### Statistical analysis

In accordance with HCUP guidelines, all analyses were performed using data weighted to produce national estimates [3]. Descriptive statistics were used to summarize patient demographics and hospital characteristics. Data elements were categorized into subgroups for analysis (Table S3). Median NIHSS scores and interquartile ranges (IQRs) were calculated for the subgroups to assess the distribution of stroke severity. The overall distribution of NIHSS scores was analyzed and interpreted by histogram analysis. NIHSS scores were clustered in accordance with the severity groupings identified by the CMS: 0, no stroke symptoms; 1–4, minor stroke; 5–15, moderate stroke; 16–20, moderate to severe stroke; and 21–42, severe stroke.

To evaluate factors influencing NIHSS reporting, a multivariable logistic regression model was performed, adjusting for patient demographics and hospital characteristics (age, sex, race, income quartile, insurance status, Elixhauser Comorbidity Index, hospital region, urban/rural hospital location, and receipt of reperfusion therapy). Adjusted odds ratios (aOR) and 95% confidence intervals (CI) were calculated to determine the likelihood of NIHSS reporting across different groups.

Propensity score matching (PSM) with a 1:1 ratio was conducted to adjust for potential confounders when comparing clinical outcomes between patients with NIHSS reported and those without. Populations were matched on the same characteristics as the logistic regression above (including All Patient Refined Diagnosis Related Groups [APR-DRG] Severity of Illness). To account for yearly trends in documentation, the weighted proportion of AIS hospitalizations with documented NIHSS scores per year was calculated. This annual reporting rate was then standardized by subtracting the median and dividing by the IQR across all study years, leaving a covariate interpretable as the change in log-odds per 1-IQR increase in a given year’s NIHSS reporting rate. This was included as a continuous covariate scaled per interquartile range in the multivariable logistic regression models. Matched cohorts were analyzed to calculate aORs and 95% CIs for routine discharge and inpatient mortality. Model discrimination was assessed using receiver operating characteristic (ROC) analysis, including the area under the curve (AUC).

NIHSS reporting was tracked over time, capturing the percentage of AIS hospitalizations every month with reported NIHSS scores. This same analysis was conducted on identified thrombectomy centers (defined as all hospitals having performed EVT at least once within a year). All statistical analyses were performed using RStudio (version 2024.09.0 + 375).

### Missing data

Missing data was identified in 5.0% of patient demographic information. This data was handled using multiple imputation with 5 iterations as per the HCUP’s recommendations for managing missing data [16].

## Results

A total of 4,558,909 AIS hospitalizations were identified from May 2016 through December 2022. Overall, NIHSS scores were reported in 1,930,880 hospitalizations (42.4%); among these patients, 937,725 (48.6%) were women, and the median age (IQR) was 70 years (60–80) (Table 1).

**Table 1:** Demographics and distribution of NIHSS scores by patient subgroup

Variable	Category	All AIS Hospitalizations (%)	Patients with NIHSS Reported (%)	Median (IQR) NIHSS Score
# of Patients		4,558,909	1,930,880	4 (2–10)
Age	< 18	16,060 (0.4)	1515 (0.1)	5 (1–12)
	18–39	131,915 (2.9)	54,075 (2.8)	3 (1–8)
	40–59	914,280 (20.1)	403,925 (20.9)	4 (1–8)
	60–79	2,233,054 (49.0)	951,795 (49.3)	4 (1–9)
	80 +	1,263,600 (27.7)	519,570 (26.9)	5 (2–13)
Sex	Male	2,317,265 (50.8)	993,155 (51.4)	4 (1–8)
	Female	2,241,644 (49.2)	937,725 (48.6)	4 (2–11)
Race	White	3,070,524 (67.4)	1,311,285 (67.9)	4 (1–9)
	Black	802,120 (17.6)	335,275 (17.4)	4 (2–10)
	Hispanic	394,905 (8.7)	162,190 (8.4)	4 (2–10)
	Asian or Pacific Islander	143,220 (3.1)	61,065 (3.2)	4 (2–11)
	Native American	23,205 (0.5)	8630 (0.4)	4 (2–10)
	Other	124,935 (2.7)	52,435 (2.7)	4 (2–11)
Income	Quartile 1	1,420,154 (31.2)	577,055 (29.9)	4 (2–10)
	Quartile 2	1,198,060 (26.3)	504,840 (26.1)	4 (2–9)
	Quartile 3	1,072,670 (23.5)	467,525 (24.2)	4 (1–9)
	Quartile 4	868,025 (19.0)	381,460 (19.8)	4 (1–9)
Insurance	Medicare	2,913,449 (63.9)	1,209,745 (62.7)	4 (2–10)
	Medicaid	475,755 (10.4)	192,925 (10.0)	4 (2–10)
	Private	859,220 (18.8)	387,655 (20.1)	3 (1–7)
	Self-Pay	178,780 (3.9)	83,000 (4.3)	3 (1–8)
	No Charge	12,775 (0.3)	6870 (0.4)	3 (1–7)
	Other	118,930 (2.6)	50,685 (2.6)	4 (2–9)
NIHSS Severity	No Stroke Symptoms	245,885 (5.4)	245,885 (12.7)	0
	Minor	795,450 (17.4)	795,450 (41.2)	2 (1–3)
	Moderate	617,635 (13.5)	617,635 (32.0)	8 (6–11)
	Moderate to Severe	120,530 (2.6)	120,530 (6.2)	18 (17–19)
	Severe	151,380 (3.3)	151,380 (7.8)	25 (22–28)
	Not reported	2,628,029 (57.6)	–	–
Hospital Location	New England	197,025 (4.3)	83,705 (4.3)	4 (1–9)
	Middle Atlantic	587,040 (12.9)	239,290 (12.4)	4 (2–9)
	East North Central	676,570 (14.8)	310,950 (16.1)	4 (2–10)
	West North Central	299,130 (6.6)	146,445 (7.6)	4 (2–10)
	South Atlantic	1,023,200 (22.4)	434,045 (22.5)	4 (1–9)
	East South Central	368,705 (8.1)	158,660 (8.2)	4 (2–10)
	West South Central	543,605 (11.9)	222,080 (11.5)	4 (1–9)
	Mountain	260,475 (5.7)	100,590 (5.2)	4 (1–9)
	Pacific	603,159 (13.2)	235,115 (12.2)	4 (2–11)
Teaching Status	Rural	328,640 (7.2)	90,715 (4.7)	4 (1–8)
	Urban Nonteaching	823,960 (18.1)	301,725 (15.6)	3 (1–8)
	Urban Teaching	3,406,309 (74.7)	1,538,440 (79.7)	4 (2–10)
Hospital Bed Size	Small	818,934 (18.0)	315,400 (16.3)	3 (1–7)
	Medium	1,298,924 (28.5)	522,870 (27.1)	4 (1–9)
	Large	2,441,051 (53.5)	1,092,610 (56.6)	4 (2–11)
Urban/Rural Classification	Central counties of metro areas of > = 1 million population	1,280,710 (28.1)	561,135 (29.1)	4 (2–10)
	Fringe counties of metro areas of > = 1 million population	1,094,689 (24.0)	475,020 (24.6)	4 (1–9)
	Counties in metro areas of 250,000–999,999 population	963,880 (21.1)	420,265 (21.8)	4 (2–9)
	Counties in metro areas of 50,000–249,999 population	440,400 (9.7)	174,505 (9.0)	4 (2–9)
	Micropolitan counties	440,770 (9.7)	172,295 (8.9)	4 (2–10)
	Not metropolitan or micropolitan counties	338,460 (7.4)	127,660 (6.6)	5 (2–11)
APR-DRG Risk of Mortality	No class specified	65 (0.0)	15 (0.0)	2 (0–13)
	Minor likelihood of dying	1,021,940 (22.4)	527,070 (27.3)	3 (1–5)
	Moderate likelihood of dying	1,360,994 (29.9)	651,250 (33.7)	3 (1–7)
	Major likelihood of dying	988,565 (21.7)	401,220 (20.8)	5 (2–11)
	Extreme likelihood of dying	1,187,345 (26.0)	351,325 (18.2)	12 (4–21)
Treatment Group	Neither	3,928,299 (86.2)	1,510,340 (78.2)	3 (1–7)
	IV Thrombolysis alone	421,725 (9.3)	272,500 (14.1)	6 (3–11)
	EVT without IV Thrombolysis	131,655 (2.9)	90,660 (4.7)	15 (9–21)
	IV Thrombolysis + EVT	77,230 (1.7)	57,380 (3.0)	16 (10–21)
Discharge Disposition	Routine Discharge	1,468,159 (32.2)	692,650 (35.9)	2 (1–4)
	Transfer to Short-term Hospital	148,360 (3.3)	49,605 (2.6)	6 (2–15)
	Other Transfer	1,844,515 (40.5)	772,490 (40.0)	6 (3–13)
	Home Health Care	720,575 (15.8)	311,160 (16.1)	3 (1–7)
	Against Medical Advice (AMA)	50,105 (1.1)	22,220 (1.2)	3 (1–6)
	Inpatient Death	325,360 (7.1)	82,600 (4.3)	19 (11–26)
	Discharge Alive, Destination Unknown	1835 (0.0)	155 (0.0)	19 (11–27)
Comorbidity Burden	Elixhauser Comorbidity Index (Mean and SD)	4.46 (2.13)	4.34 (2.02)	–
	Length of Stay (Mean and SD)	6.93 (10.59)	5.53 (7.13)	–
	Total Charges (Mean and SD)	102,747.41 (191,095.04)	88,177.04 (116,293.32)	–

Infarct location and stroke mechanism were further characterized. Among hospitalizations with reported NIHSS scores, the most frequently coded infarct territory was a middle cerebral artery (28.0%), followed by unspecified small artery (7.1%), carotid artery (6.0%), posterior cerebral artery (4.7%), and cerebellar artery (1.2%). An unspecified arterial territory was coded in 47.3% of these cases. With respect to stroke mechanism, embolic stroke was reported in 21.4% of hospitalizations with documented NIHSS scores, while thrombosis was reported in 6.4% and the remaining 72.1% were unspecified. The distribution of infarct location and mechanism differed significantly between hospitalizations with and without reported NIHSS scores (all p < 0.001 per the Rao-Scott correction of the Pearson chi-square test) (Table S4).

**Fig. 1:**
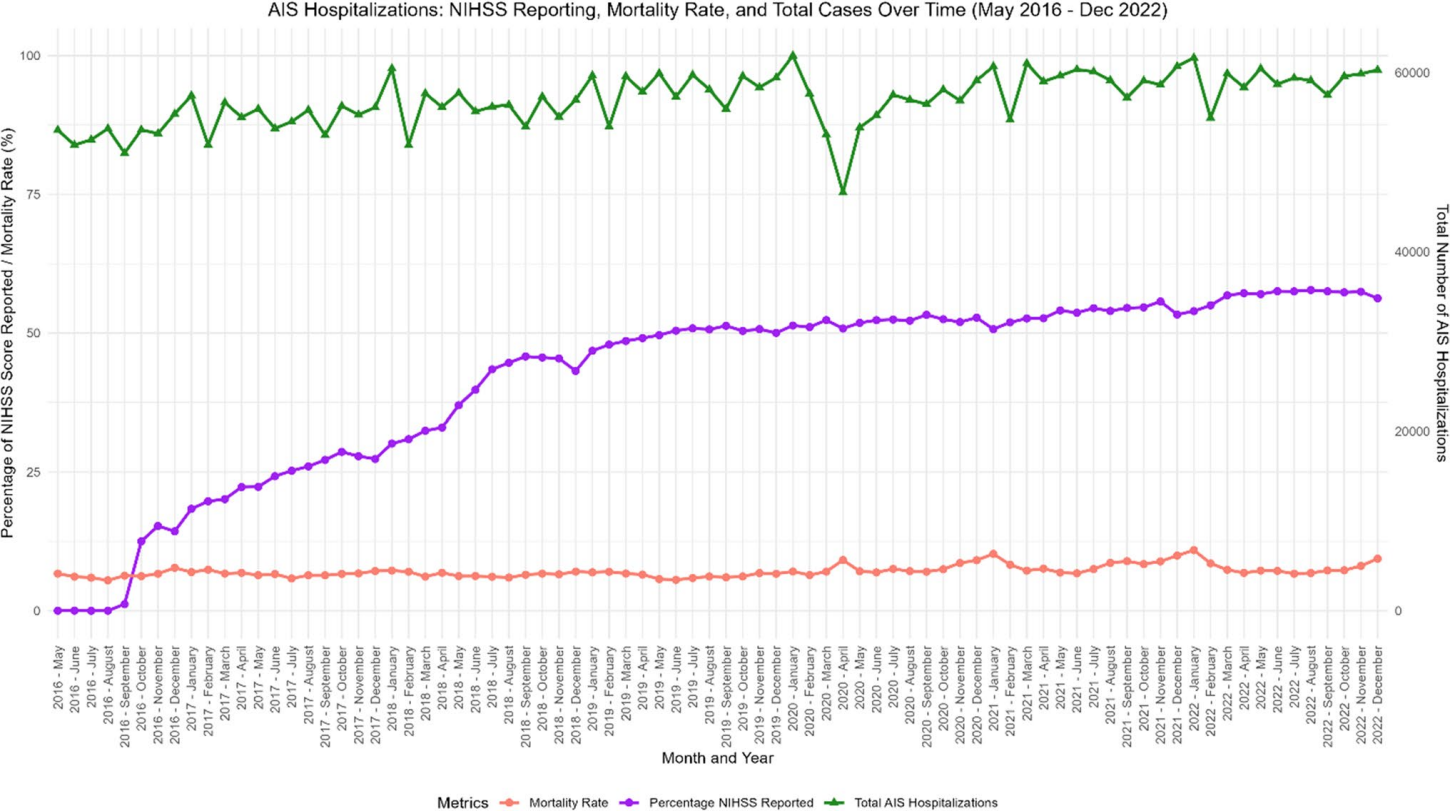
NIHSS reporting, mortality rate, and total ais hospitalizations over time (by month)

Total AIS hospitalizations increased from approximately 50,000/month to 60,000/month over the study period. There was a consistent drop in reported AIS hospitalizations at the start of every year; the most significant being from 61,885 hospitalizations in January 2020 to 46,620 in April 2020 (Table S2). NIHSS reporting rates steadily increased throughout the study period, growing from 14.33% in December 2016 to 56.27% in December 2022. The reporting rate surpassed 50% in June 2019 but plateaued through December 2022. A subgroup analysis on thrombectomy centers revealed that the reporting rate rose from 20.64% in December 2016 to 61.78% in December 2022 (Table S5). The reporting rate surpassed 50% in July 2018, almost a year before the overall dataset, but plateaued around 60% soon afterwards (Fig. 1, Figure S1).

**Fig. 2:**
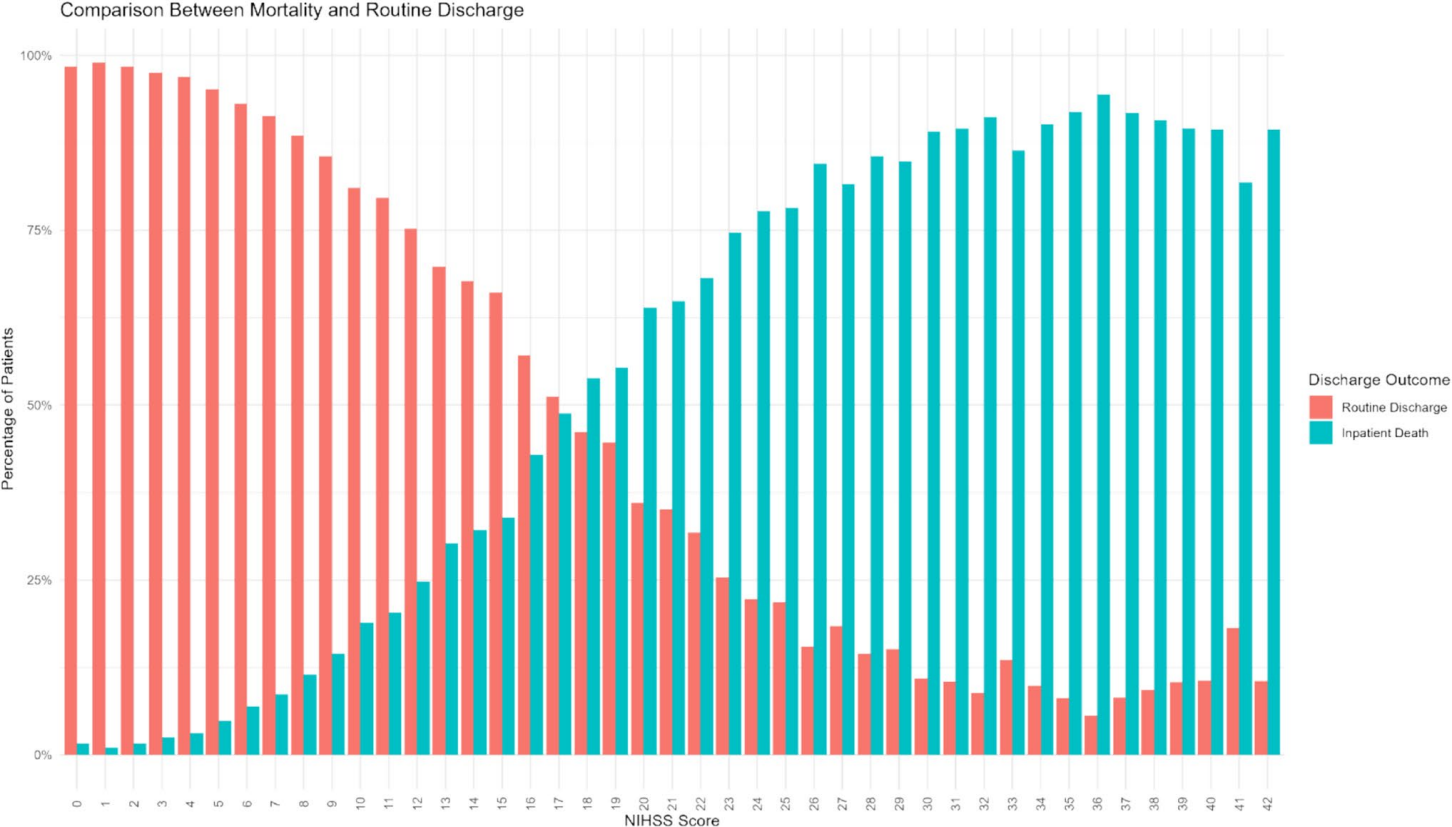
Comparison between mortality and routine discharge by NIHSS score

The median NIHSS score (IQR) was 4 (2–10). The distribution of NIHSS scores by severity grouping was: 245,885 hospitalizations (12.7%) had no stroke symptoms; 795,450 (41.2%) had minor stroke; 617,635 (32.0%) had moderate stroke; 120,530 (6.2%) had moderate to severe stroke; and 151,380 (7.8%) had severe stroke (Table 1). The histogram of NIHSS score distribution demonstrated a positively skewed distribution, with most patients presenting with lower NIHSS scores (Figure S2). The odds of inpatient mortality increased as NIHSS scores increased, while the odds of routine discharge and inpatient mortality were equivalent when a patient’s NIHSS score was 17–18 (Fig. 2).

NIHSS scores were mostly uniform in terms of median and IQR, mainly differing between discharge disposition and treatment group. By discharge status, median (IQR) NIHSS was: 2 (1–4) for routine discharge; 6 (2–15) for transfer to short-term hospital; 6 (3–13) for other transfer; 3 (1–7) for home health care; 3 (1–6) for against medical advice; 19 (11–26) for inpatient death; and 19 (11–27) for discharge alive, destination unknown. Regarding reperfusion therapy, median (IQR) NIHSS was: 16 (10–21) for IVT and EVT; 15 (9–21) for EVT alone; 6 (3–11) for IVT alone; and 3 (1–7) for no treatment. There were also minor variations in the median (IQR) NIHSS score depending on age, with pediatric and elderly patients having a higher median NIHSS of 5 and a wider IQR (1–12 and 2–13, respectively) compared to other age groups (Table 1).

Multivariable logistic regression analysis including patient demographics and hospital characteristics identified independent factors associated with a higher likelihood of NIHSS score reporting. These factors included male sex, White/Asian race, higher income quartile, non-federal insurance, lower comorbidity burden, hospital location in the East and West North Central zones, larger hospital size, teaching hospital status, hospital location in largely populated urban areas, and receipt of any reperfusion therapy (Table 2).

**Table 2:** Multivariable logistic regression for the impact of patient and hospital factors on NIHSS reporting

Variable	Characteristic	Odds Ratio	95% Confidence Interval	P-Value
	Age	1.004	[1.004–1.005]	** < 0.001**
Sex	Male	1 [Reference]		
	Female	0.970	[0.961–0.979]	** < 0.001**
Race/Ethnicity	White	1 [Reference]		
	Black	0.963	[0.939–0.988]	**0.004**
	Hispanic	0.960	[0.926–0.996]	**0.030**
	Asian or Pacific Islander	1.02	[0.973–1.06]	0.449
	Native American	0.914	[0.836–0.999]	**0.048**
	Other	0.939	[0.898–0.983]	**0.006**
Income Quartile	Quartile 1	1 [Reference]		
	Quartile 2	1.05	[1.02–1.07]	** < 0.001**
	Quartile 3	1.07	[1.04–1.10]	** < 0.001**
	Quartile 4	1.08	[1.04–1.12]	** < 0.001**
Insurance Status	Medicare	1 [Reference]		
	Medicaid	1.02	[1.00–1.04]	0.052
	Private	1.11	[1.09–1.13]	** < 0.001**
	Self-Pay	1.22	[1.18–1.26]	** < 0.001**
	No Charge	1.62	[1.46–1.81]	** < 0.001**
	Other	1.06	[1.01–1.10]	**0.008**
Comorbidities	Elixhauser Comorbidity Index Score	0.940	[0.937–0.944]	** < 0.001**
Hospital Location	New England	1 [Reference]		
	Middle Atlantic	0.860	[0.774–0.955]	**0.005**
	East North Central	1.17	[1.06–1.30]	**0.002**
	West North Central	1.31	[1.16–1.48]	** < 0.001**
	South Atlantic	0.999	[0.905–1.10]	0.992
	East South Central	1.08	[0.942–1.24]	0.274
	West South Central	0.941	[0.844–1.05]	0.276
	Mountain	0.787	[0.688–0.901]	** < 0.001**
	Pacific	0.808	[0.727–0.897]	** < 0.001**
Hospital Teaching Status	Rural	1 [Reference]		
	Urban Nonteaching	1.54	[1.42–1.67]	** < 0.001**
	Urban Teaching	2.11	[1.95–2.28]	** < 0.001**
Hospital Bed Size	Small	1 [Reference]		
	Medium	1.07	[1.01–1.13]	**0.012**
	Large	1.27	[1.21–1.34]	** < 0.001**
Urban/Rural Hospital Classification	Central counties of metro areas ≥ 1 M population	1 [Reference]		
	Fringe counties of metro areas ≥ 1 M population	0.957	[0.917–0.999]	**0.044**
	Counties in metro areas 250 k-999 k population	0.970	[0.920–1.02]	0.251
	Counties in metro areas 50 k-249 k population	0.846	[0.797–0.898]	** < 0.001**
	Micropolitan counties	0.9998	[0.946–1.05]	0.935
	Not metropolitan or micropolitan counties	0.876	[0.827–0.929]	** < 0.001**
Reperfusion Therapy	Neither	1 [Reference]		
	IV Thrombolysis alone	2.80	[2.74–2.86]	** < 0.001**
	EVT without IV Thrombolysis	3.31	[3.18–3.45]	** < 0.001**
	IV Thrombolysis + EVT	4.26	[4.05–4.47]	** < 0.001**

ORs represent the odds of NIHSS being reported associated with each variable. 95% CIs indicate the range within which the true OR is expected to lie with 95% confidence

After PSM, the inpatient mortality rate was significantly lower in hospitalizations with reported NIHSS scores (4.26% vs. 8.21%). Hospitalizations with a reported NIHSS had a higher likelihood of routine discharge (aOR: 1.03, 95% CI [1.01–1.04], p < 0.001) and a lower likelihood of inpatient mortality (0.650 [0.632–0.668], p < 0.001). In contrast, each IQR increase in annual NIHSS reporting rate was associated with increased inpatient mortality (1.06 [1.04–1.09], p < 0.001) and was not associated with routine discharge (0.998 [0.987–1.01], p = 0.784). Additionally, nearly all patient and hospital characteristics demonstrated significant impacts on the odds of routine discharge and inpatient mortality when compared to the reference groups (male sex, White race, lowest income quartile, Medicare insurance, hospital region in New England, urban/rural hospital classification, and no receipt of reperfusion therapy) (Table 3). When assessing model performance, the AUC for the inpatient mortality model was 0.889 (95% CI: 0.887–0.890), while the AUC for the routine discharge model was 0.777 (95% CI: 0.776–0.778).

**Table 3:** Propensity score matching results – odds ratios and 95% confidence intervals for routine discharge and inpatient mortality outcomes

		**Routine Discharge**		**Inpatient Mortality**	
Variable	Characteristic	Odds Ratio (95% Confidence Interval)	P-Value	Odds Ratio (95% Confidence Interval)	P-Value
	NIHSS Reported	1.03 (1.01–1.04)	** < 0.001**	0.650 (0.632–0.668)	** < 0.001**
	Annual NIHSS Reporting Rate [IQR Increase]	0.998 (0.987–1.01)	0.784	1.06 (1.04–1.09)	** < 0.001**
	Age	0.961 (0.961–0.962)	** < 0.001**	1.030 (1.029–1.032)	** < 0.001**
Sex	Male	1 [Reference]		1 [Reference]	
	Female	0.791 (0.783–0.800)	** < 0.001**	1.01 (0.983–1.04)	0.398
Race	White	1 [Reference]		1 [Reference]	
	Black	0.788 (0.774–0.803)	** < 0.001**	0.842 (0.810–0.876)	** < 0.001**
	Hispanic	1.03 (1.00–1.06)	**0.030**	0.936 (0.891–0.984)	**0.010**
	Asian or Pacific Islander	0.890 (0.856–0.925)	** < 0.001**	0.960 (0.889–1.04)	0.304
	Native American	1.13 (1.03–1.23)	**0.007**	1.02 (0.844–1.24)	0.803
	Other	0.921 (0.887–0.956)	** < 0.001**	1.01 (0.939–1.09)	0.724
Income Quartile	Quartile 1	1 [Reference]		1 [Reference]	
	Quartile 2	1.04 (1.03–1.06)	** < 0.001**	0.963 (0.931–0.997)	**0.032**
	Quartile 3	1.12 (1.10–1.14)	** < 0.001**	0.955 (0.919–0.993)	**0.021**
	Quartile 4	1.17 (1.15–1.19)	** < 0.001**	0.909 (0.871–0.950)	** < 0.001**
Insurance Status	Medicare	1 [Reference]		1 [Reference]	
	Medicaid	1.08 (1.05–1.10)	** < 0.001**	1.29 (1.23–1.36)	** < 0.001**
	Private	1.38 (1.36–1.41)	** < 0.001**	1.44 (1.37–1.52)	** < 0.001**
	Self-Pay	2.25 (2.17–2.32)	** < 0.001**	1.94 (1.80–2.10)	** < 0.001**
	No Charge	2.71 (2.43–3.02)	** < 0.001**	1.50 (1.15–1.94)	**0.002**
	Other	1.25 (1.20–1.30)	** < 0.001**	2.68 (2.44–2.94)	** < 0.001**
Comorbidities	Elixhauser Comorbidity Index Score	0.864 (0.860–0.867)	** < 0.001**	0.971 (0.964–0.978)	** < 0.001**
Hospital Region	New England	1 [Reference]		1 [Reference]	
	Middle Atlantic	1.43 (1.37–1.49)	** < 0.001**	0.879 (0.803–0.962)	**0.006**
	East North Central	1.82 (1.75–1.90)	** < 0.001**	0.744 (0.691–0.801)	** < 0.001**
	West North Central	1.87 (1.78–1.96)	** < 0.001**	0.801 (0.733–0.875)	** < 0.001**
	South Atlantic	1.59 (1.53–1.66)	** < 0.001**	0.648 (0.600–0.700)	** < 0.001**
	East South Central	1.80 (1.71–1.89)	** < 0.001**	0.835 (0.758–0.919)	** < 0.001**
	West South Central	1.66 (1.59–1.74)	** < 0.001**	0.685 (0.626–0.748)	** < 0.001**
	Mountain	1.83 (1.74–1.93)	** < 0.001**	0.681 (0.616–0.752)	** < 0.001**
	Pacific	1.59 (1.51–1.66)	** < 0.001**	0.966 (0.893–1.04)	0.380
Teaching Status	Rural	1 [Reference]		1 [Reference]	
	Urban Nonteaching	1.05 (1.01–1.09)	**0.008**	0.859 (0.789–0.936)	** < 0.001**
	Urban Teaching	1.17 (1.13–1.21)	** < 0.001**	0.982 (0.907–1.06)	0.645
Bed Size	Small	1 [Reference]		1 [Reference]	
	Medium	1.04 (1.02–1.06)	**0.001**	1.16 (1.10–1.23)	** < 0.001**
	Large	1.08 (1.06–1.11)	** < 0.001**	1.18 (1.13–1.24)	** < 0.001**
Urban/Rural Hospital Location	Central counties of metro areas ≥ 1 M population	1 [Reference]		1 [Reference]	
	Fringe counties of metro areas ≥ 1 M population	1.02 (1.00–1.04)	**0.031**	1.05 (1.01–1.10)	**0.014**
	Counties in metro areas 250 k-999 k population	1.03 (1.01–1.06)	**0.006**	1.13 (1.08–1.18)	** < 0.001**
	Counties in metro areas 50 k-249 k population	1.10 (1.07–1.13)	** < 0.001**	1.25 (1.18–1.32)	** < 0.001**
	Micropolitan counties	1.07 (1.04–1.11)	** < 0.001**	1.29 (1.21–1.37)	** < 0.001**
	Not metropolitan or micropolitan counties	1.10 (1.07–1.13)	** < 0.001**	1.32 (1.24–1.41)	** < 0.001**
Reperfusion Therapy	Neither	1 [Reference]		1 [Reference]	
	IV Thrombolysis alone	1.26 (1.24–1.28)	** < 0.001**	1.05 (1.01–1.09)	**0.009**
	EVT without IV Thrombolysis	0.654 (0.627–0.681)	** < 0.001**	1.30 (1.24–1.36)	** < 0.001**
	IV Thrombolysis + EVT	0.880 (0.842–0.919)	** < 0.001**	1.18 (1.12–1.26)	** < 0.001**
	Area Under the Curve	0.777 (0.776–0.778)		0.889 (0.887–0.890)	

ORs represent the odds of Routine Discharge and Inpatient Mortality associated with each variable after propensity score matching. 95% CIs indicate the range within which the true OR is expected to lie with 95% confidence

## Discussion

This study offers a comprehensive longitudinal analysis of NIHSS reporting rates in a nationally representative database from 2016 to 2022. Our findings reveal a significant increase in NIHSS reporting over time, disparities in reporting practices, and associations between NIHSS reporting and inpatient outcomes.

Our findings build upon existing research by providing a longitudinal perspective, highlighting an increase and eventual plateau in NIHSS reporting. We confirm that 14.33% of AIS hospitalizations had reported NIHSS scores in December 2016 yet also demonstrate a substantial rise in reporting to 56.27% by December 2022 [28]. We also revealed distinct phases in the adoption of NIHSS reporting; early adopters rapidly increased the reporting rate in the initial period post-2016, however, reporting rates began to plateau around 50% in July 2019. This asymptotic slowing may be partially attributed to the COVID-19 pandemic, which disrupted healthcare systems and potentially hindered further improvements in NIHSS reporting [33]. Additionally, inherent systemic barriers such as limited training, resource constraints, and variability in institutional priorities likely contributed to the stagnation, preventing NIHSS reporting from reaching 100% compliance despite mounting policy pressures [5, 13].

A major focus of this study was the impact of patient-level and hospital-level characteristics on the reporting of NIHSS scores. Our results indicated that patients identified as female, non-White/Asian race, belonging to lower income quartiles (ZIP-based income quartile), having higher comorbidity burden, not receiving any reperfusion therapy or having federal insurance were less likely to have their NIHSS scores reported. Other factors associated with lower likelihood of NIHSS reporting were hospital location in the Middle Atlantic, Mountain, and Pacific zones, hospital location in less populated areas, and smaller sized hospitals. This contrasts with other studies which associated male sex with decreased reporting likelihood, while supporting that highly populated metropolitan areas are more likely to report scores [28, 30]. Prior studies have also shown that hospitals which are teaching hospitals, stroke certified, higher volume, and possessing ICU capacity were more likely to report NIHSS scores [30]. These disparities potentially reflect socioeconomic and systemic factors that may impede consistent NIHSS utilization. For instance, lower-income patients or those with federal insurance may receive care at hospitals with fewer resources or less specialized stroke care protocols, leading to reduced NIHSS reporting. Similarly, hospitals with fewer resources may lack the necessary training or infrastructure to consistently implement NIHSS assessments.

A significant finding of our study is that NIHSS reporting is associated with lower odds of inpatient mortality and higher likelihood of routine discharge. This association suggests that NIHSS reporting may serve as a proxy for higher-quality care. Possible explanations include more thorough clinical assessments, better adherence to treatment protocols, enhanced monitoring facilitated by standardized severity grading, and increased awareness of stroke severity among healthcare providers. Furthermore, NIHSS reporting may enable more precise risk stratification, allowing for individualized interventions that improve survival odds. Our analysis also shows significantly greater reporting in hospitalizations involving reperfusion therapy for AIS; thus, it is unlikely that the improved survival rates with reported NIHSS are a result of decreased documentation in severe cases. Additionally, although NIHSS reporting increased substantially over the study period, higher annual reporting rates were not associated with improved outcomes. This suggests that improvements in outcomes are not explained by increasing NIHSS reporting rates alone, but rather that individual NIHSS documentation may reflect higher-quality, guideline-directed stroke care. The observed association between higher annual reporting rates and mortality likely reflects residual confounding at the year level, where years with higher NIHSS documentation had increased case complexity and a higher proportion of severe strokes.

The NIHSS score distribution in our study was positively skewed, with the majority of patients presenting with lower severity scores. With 41.2% of individuals with reported NIHSS falling under a “minor” stroke range and a median NIHSS of 4, our determined distribution closely matches those available from prior studies such as the Get with the Guidelines-Stroke registry and the Cincinnati/Northern Kentucky Stroke Study [26, 28, 35]. A notable difference in our distribution, however, is the relatively large proportion of patients with a reported NIHSS score of zero (12.7%). The presence of these records suggests presenting symptoms and history that warranted ruling out AIS, potentially reflecting improved public awareness of stroke symptoms and appropriately seeking medical care.

When comparing mortality and routine discharge among each NIHSS score, our analysis shows an inverse relationship between routine discharge and mortality. This finding aligns with existing literature linking NIHSS scores with patient outcomes [20]. Notably, the convergence of odds for routine discharge and inpatient mortality at NIHSS scores 17–18 represents a critical threshold for clinical decision-making regarding treatment. This phenomenon requires further investigation as to the clinical or disease-inherent parameters that cause this ambiguity in outcome.

The substantial decline in AIS hospitalizations from January-April 2020 coincides with the onset of the COVID-19 pandemic, possibly reflecting patients' reluctance to seek hospital care due to fear of infection, disruptions in healthcare services, or true reductions in stroke incidence related to lifestyle changes during the pandemic [7, 14, 32, 33]. While hospitals were facing resource/staffing shortages and increased patient loads, it is important to note that NIHSS reporting rates did not decrease during this time period, highlighting consistent data collection and thorough documentation efforts. This aligns with prior research showing the maintenance of acute stroke care throughout the pandemic era [4, 27]. Moreover, our study detected a consistent drop in reported AIS hospitalizations at the start of each year. It is unclear whether this is a result of seasonal variations in stroke incidence, systemic issues in NIS data sampling, or a temporal disruption in healthcare services.

Our findings highlight the critical role of NIHSS reporting in standardizing stroke severity assessment and its association with improved outcomes in AIS. To enhance NIHSS utilization, hospitals should continue to invest in training and certification programs, integrate NIHSS assessments into electronic health record systems, and adopt standardized protocols for stroke care. Policymakers may consider implementing additional incentives or mandates to ensure consistent NIHSS reporting, particularly in hospitals serving disadvantaged populations.

## Limitations

This study has several limitations inherent to the use of a large national administrative database. First, reliance on ICD-10-CM/PCS codes to identify AIS hospitalizations, NIHSS scores, and interventions may introduce potential coding errors and variability across institutions. Previous studies have demonstrated inconsistent coding accuracy, potentially affecting the identification of cases and procedures [12, 15, 19, 23, 31]. Furthermore, although ICD-10-CM codes permit limited classification by infarct territory and coded stroke type, the NIS does not contain etiologic stroke subtype information (e.g., lacunar, large-artery atherosclerotic, or cardioembolic stroke), precluding mechanistic subtype analysis. Second, the NIS does not specify the timing of NIHSS score documentation within each hospitalization. Although CMS guidelines state, “At a minimum, report the initial score documented. If desired, a facility may choose to capture multiple stroke scale scores,” the dataset does not indicate whether the reported NIHSS reflects the admission score, a subsequent assessment, or a discharge score [11]. Discharge NIHSS scores are not explicitly identifiable in the NIS, thus we cannot distinguish between neurologic status at presentation versus at hospital discharge. Given that NIHSS scores may change substantially over the course of hospitalization, this limitation may affect the interpretation of NIHSS in relation to patient outcomes. Third, multiple NIHSS scores could have been reported for the same hospitalization. Since the dataset does not specify the sequence of NIHSS scores, we used the highest NIHSS score reported for each hospitalization in our analysis. While this approach assumes that the highest score reflects the most severe neurological status, it may not accurately represent the initial presentation if documentation practices varied. Fourth, it is possible that NIHSS scores were recorded in patients’ medical records but not included in the billing data from which the NIS is derived, leading to potential missingness that cannot be identified or accounted for in our analysis. Additionally, selection biases inherent in administrative databases may affect our findings. Unmeasured confounding variables not captured in the database, including baseline functional status, clinical nuances, hospital protocols, and provider practices, could influence both NIHSS reporting and patient outcomes. In particular, the NIS does not capture pre-stroke functional status (e.g., pre-stroke modified Rankin Scale), which is an important determinant of discharge disposition and mortality. Although we adjusted for a comprehensive set of patient demographics, comorbidity burden using the Elixhauser Comorbidity Index, and stroke severity using APR-DRG Severity of Illness, residual confounding related to unmeasured baseline functional impairment may persist. Despite these limitations, the use of nationwide data enhances the generalizability of our findings. Leveraging a comprehensive national database mitigates the potential effects of hospital-specific biases and provides valuable insights into NIHSS reporting and its associated outcomes on a national scale.

## Conclusions

Since the beginning of NIHSS score reporting in the NIS, this study observed a significant increase in its documentation among AIS hospitalizations in the United States, from 14.33% in December 2016 to 56.27% in December 2022. The likelihood of reporting was dependent on patient demographics and hospital characteristics, highlighting persistent disparities in stroke care. The presence of a reported score was also associated with higher odds of routine discharge and lower inpatient mortality, indicating nationwide variability in the quality of stroke care. Further research is warranted to explore the underlying reasons for variations in NIHSS documentation and to develop strategies that encourage widespread adoption of this clinical assessment tool.

## Supplementary Information

Below is the link to the electronic supplementary material.

Supplementary file1 (DOCX 17 KB)

Supplementary file2 (DOCX 20 KB)

Supplementary file3 (DOCX 15 KB)

Supplementary file4 (DOCX 16 KB)

Supplementary file5 (DOCX 20 KB)

Supplementary file6 (JPEG 1258 KB)

Supplementary file7 (JPEG 624 KB)

Supplementary file8 (DOCX 32 KB)

## Data Availability

Data not available due to legal restrictions. The data that support the findings of this study are available from the Healthcare Cost and Utilization Project (HCUP) National Inpatient Sample but restrictions apply to their availability. These data were used under license for the current study and therefore cannot be shared by the authors. Researchers may obtain the data directly from HCUP after completing the required Data Use Agreement and purchasing the dataset through the HCUP website (https://hcup-us.ahrq.gov/).

## References

[1] Adams HP Jr, Davis PH, Leira EC et al (1999) Baseline NIH Stroke Scale score strongly predicts outcome after stroke: A report of the Trial of Org 10172 in Acute Stroke Treatment (TOAST). Neurology 53(1):126–131. 10.1212/wnl.53.1.126 10408548 10.1212/wnl.53.1.126

[2] Agency for Healthcare Research and Quality. NIS Overview. Healthcare Cost and Utilization Project. https://hcup-us.ahrq.gov/nisoverview.jsp. Accessed October 22, 2024.

[3] Agency for Healthcare Research and Quality. Producing National HCUP Estimates - Accessible Version. Healthcare Cost and Utilization Project. https://hcup-us.ahrq.gov/tech_assist/nationalestimates/508_course/508course_2018.jsp#weights. Accessed October 22, 2024.

[4] Altersberger VL, Stolze LJ, Heldner MR et al (2021) Maintenance of acute stroke care service during the COVID-19 pandemic lockdown. Stroke 52(5):1693–1701. 10.1161/STROKEAHA.120.032176 33793320 10.1161/STROKEAHA.120.032176 PMC8078117

[5] Baatiema L, Otim ME, Mnatzaganian G, de-Graft Aikins A, Coombes J, Somerset S (2017) Health professionals’ views on the barriers and enablers to evidence-based practice for acute stroke care: a systematic review. Implement Sci 12(1):74. 10.1186/s13012-017-0599-3 28583164 10.1186/s13012-017-0599-3 PMC5460544

[6] Berge E, Whiteley W, Audebert H et al (2021) European stroke organisation (ESO) guidelines on intravenous thrombolysis for acute ischaemic stroke. Eur Stroke J 6(1):I–LXII. 10.1177/2396987321989865 33817340 10.1177/2396987321989865 PMC7995316

[7] Bres Bullrich M, Fridman S, Mandzia JL et al (2020) COVID-19: stroke admissions, emergency department visits, and prevention clinic referrals. Can J Neurol Sci 47(5):693–696. 10.1017/cjn.2020.101 32450927 10.1017/cjn.2020.101 PMC7324648

[8] Brott T, Adams HP Jr, Olinger CP et al (1989) Measurements of acute cerebral infarction: a clinical examination scale. Stroke 20(7):864–870. 10.1161/01.str.20.7.864 2749846 10.1161/01.str.20.7.864

[9] Centers for Medicare & Medicaid Services. 2022 Condition-Specific Mortality Measures: Updates and Specifications Report. CMS.gov. https://www.cms.gov/files/document/2022-condition-specific-mortality-measures-updates-and-specifications-report.pdf. Published April 2022. Accessed November 15, 2024.

[10] Centers for Medicare & Medicaid Services. 2018 ICD-10-CM Official Guidelines for Coding and Reporting. CMS.gov. https://www.cms.gov/Medicare/Coding/ICD10/Downloads/2018-ICD-10-CM-Coding-Guidelines.pdf. Published 2018. Accessed November 15, 2024.

[11] Centers for Medicare & Medicaid Services. FY 2024 ICD-10-CM Official Guidelines for Coding and Reporting. CMS.gov. https://www.cms.gov/files/document/fy-2024-icd-10-cm-coding-guidelines-updated-02/01/2024.pdf. Published 2024. Accessed October 24, 2024.

[12] Comer AR, Templeton E, Glidden M et al (2023) National institutes of health stroke scale (NIHSS) scoring inconsistencies between neurologists and emergency room nurses. Front Neurol 13:1093392. 10.3389/fneur.2022.1093392 36712449 10.3389/fneur.2022.1093392 PMC9875120

[13] Cormican A, Hirani SP, McKeown E (2023) Healthcare professionals’ perceived barriers and facilitators of implementing clinical practice guidelines for stroke rehabilitation: a systematic review. Clin Rehabil 37(5):701–712. 10.1177/02692155221141036 36475911 10.1177/02692155221141036 PMC10041573

[14] Esenwa C, Parides MK, Labovitz DL (2020) The effect of COVID-19 on stroke hospitalizations in New York City. J Stroke Cerebrovasc Dis 29(10):105114. 10.1016/j.jstrokecerebrovasdis.2020.105114 32912527 10.1016/j.jstrokecerebrovasdis.2020.105114 PMC7355321

[15] Horsky J, Drucker EA, Ramelson HZ (2018) Accuracy and Completeness of Clinical Coding Using ICD-10 for Ambulatory Visits. AMIA Annu Symp Proc 2017:912–920 (**Published 2018 Apr 16**) 29854158 PMC5977598

[16] Houchens R. Missing Data Methods for the NIS and the SID. 2015. HCUP Methods Series Report # 2015–01 ONLINE. January 22, 2015. U.S. Agency for Healthcare Research and Quality. Available: http://www.hcup-us.ahrq.gov/reports/methods/methods.jsp.

[17] Jain A, Van Houten D, Sheikh L (2016) Retrospective Study on National Institutes of Health Stroke Scale as a Predictor of Patient Recovery After Stroke. J Cardiovasc Nurs 31(1):69–72. 10.1097/JCN.0000000000000198 25325366 10.1097/JCN.0000000000000198

[18] Kamel H, Liberman AL, Merkler AE et al (2023) Validation of the *International Classification of Diseases, Tenth Revision* Code for the National Institutes of Health Stroke Scale Score. Circ Cardiovasc Qual Outcomes 16(3):e009215. 10.1161/CIRCOUTCOMES.122.009215 36862375 10.1161/CIRCOUTCOMES.122.009215 PMC10237010

[19] Kokotailo RA, Hill MD (2005) Coding of stroke and stroke risk factors using international classification of diseases, revisions 9 and 10. Stroke 36(8):1776–1781. 10.1161/01.STR.0000174293.17959.a1 16020772 10.1161/01.STR.0000174293.17959.a1

[20] Kumar A, Roy I, Bosch PR et al (2022) Medicare claim-based National Institutes of Health stroke scale to predict 30-day mortality and hospital readmission. J Gen Intern Med 37(11):2719–2726. 10.1007/s11606-021-07162-0 34704206 10.1007/s11606-021-07162-0 PMC9411458

[21] Lyden P, Raman R, Liu L, Emr M, Warren M, Marler J (2009) National Institutes of Health Stroke Scale certification is reliable across multiple venues. Stroke 40(7):2507–2511. 10.1161/STROKEAHA.108.532069 19520998 10.1161/STROKEAHA.108.532069 PMC2726278

[22] New York State Department of Health. NYSDOH Stroke Services Guidance Document. New York State Department of Health. https://www.health.ny.gov/diseases/cardiovascular/stroke/designation/docs/nysdoh_stroke_guidance_document. Published March 1, 2023. Accessed November 15, 2024.

[23] O’Malley KJ, Cook KF, Price MD, Wildes KR, Hurdle JF, Ashton CM (2005) Measuring diagnoses: ICD code accuracy. Health Serv Res 40(5 Pt 2):1620–1639. 10.1111/j.1475-6773.2005.00444.x 16178999 10.1111/j.1475-6773.2005.00444.x PMC1361216

[24] Powers WJ, Rabinstein AA, Ackerson T, et al. Guidelines for the Early Management of Patients With Acute Ischemic Stroke: 2019 Update to the 2018 Guidelines for the Early Management of Acute Ischemic Stroke: A Guideline for Healthcare Professionals From the American Heart Association/American Stroke Association [published correction appears in Stroke. 2019 Dec;50(12):e440-e441. 10.1161/STR.0000000000000215 10.1161/STR.0000000000000215 31765293

[25] Reeves MJ, Smith EE, Fonarow GC et al (2015) Variation and Trends in the Documentation of National Institutes of Health Stroke Scale in GWTG-Stroke Hospitals. Circ Cardiovasc Qual Outcomes 8(6 Suppl 3):S90–S98. 10.1161/CIRCOUTCOMES.115.001775 26515215 10.1161/CIRCOUTCOMES.115.001775

[26] Reeves M, Khoury J, Alwell K et al (2013) Distribution of National Institutes of Health stroke scale in the Cincinnati/Northern Kentucky Stroke Study. Stroke 44(11):3211–3213. 10.1161/STROKEAHA.113.002881 24003048 10.1161/STROKEAHA.113.002881 PMC4632977

[27] Romoli M, Eusebi P, Forlivesi S et al (2021) Stroke network performance during the first COVID-19 pandemic stage: a meta-analysis based on stroke network models. Int J Stroke 16(7):771–783. 10.1177/17474930211041202 34427480 10.1177/17474930211041202 PMC8521356

[28] Saber H, Saver JL (2020) Distributional Validity and Prognostic Power of the National Institutes of Health Stroke Scale in US Administrative Claims Data. JAMA Neurol 77(5):606–612. 10.1001/jamaneurol.2019.5061 32065612 10.1001/jamaneurol.2019.5061 PMC7042858

[29] Schlegel D, Kolb SJ, Luciano JM et al (2003) Utility of the NIH Stroke Scale as a predictor of hospital disposition. Stroke 34(1):134–137. 10.1161/01.str.0000048217.44714.02 12511764 10.1161/01.str.0000048217.44714.02

[30] Stein LK, Ortiz E, Nandwani J, Dhamoon MS (2024) National Institutes of Health Stroke Scale Reporting in Medicare Claims Data: Reporting in the First 3 Years. Circ Cardiovasc Qual Outcomes 17(4):e010388. 10.1161/CIRCOUTCOMES.123.010388 38597090 10.1161/CIRCOUTCOMES.123.010388

[31] Taha M, Habib M, Lomachinsky V et al (2024) Evaluating the concordance between International Classification of Diseases, Tenth Revision Code and stroke severity as measured by the National Institutes of Health Stroke Scale. BMJ Neurol Open 6(2):e000831. 10.1136/bmjno-2024-000831 39363950 10.1136/bmjno-2024-000831 PMC11448239

[32] Uchino K, Kolikonda MK, Brown D et al (2020) Decline in stroke presentations during COVID-19 surge. Stroke 51(8):2544–2547. 10.1161/STROKEAHA.120.030331 32716818 10.1161/STROKEAHA.120.030331 PMC7309646

[33] Yang Q, Tong X, Coleman King S, Olivari BS, Merritt RK (2021) Stroke hospitalizations before and during COVID-19 pandemic among Medicare beneficiaries in the United States. Stroke 52(11):3586–3601. 10.1161/STROKEAHA.121.034562 34320816 10.1161/STROKEAHA.121.034562 PMC8547589

[34] Zachrison KS, Li S, Reeves MJ et al (2021) Strategy for reliable identification of ischaemic stroke, thrombolytics and thrombectomy in large administrative databases. Stroke Vasc Neurol 6(2):194–200. 10.1136/svn-2020-000533 33177162 10.1136/svn-2020-000533 PMC8258073

[35] Ziaeian B, Xu H, Matsouaka RA et al (2022) US surveillance of acute ischemic stroke patient characteristics, care quality, and outcomes for 2019. Stroke 53(11):3386–3393. 10.1161/STROKEAHA.122.039098 35862201 10.1161/STROKEAHA.122.039098 PMC9613506

