# Supplementary material for: Trends and disparities in NIHSS reporting and outcomes in acute ischemic stroke hospitalizations: A retrospective cross-sectional study
Source: Acta Neurochir (Wien). 2026 Apr 21;168(1):126. doi: 10.1007/s00701-026-06870-y (PMC13234067; doi:10.1007/s00701-026-06870-y)
Supplement: Supplementary file 1 — Supplementary file1 (DOCX 17 KB) [file 701_2026_6870_MOESM1_ESM.docx]

**Table S1.** Overview of ICD-10-CM Codes and National Inpatient Sample Data Elements for Identification of Diagnosis, Procedure, and Patient Information/Outcomes

| **Variable** | **International Classification of Diseases, 10th Revision, Clinical Modification and Procedure Codes** | **NIS Data Element** |
| --- | --- | --- |
| Acute Ischemic Stroke | I63.00, I63.011, I63.012, I63.013, I63.019, I63.02, I63.031, I63.032, I63.033, I63.039, I63.09, I63.10, I63.111, I63.112, I63.113, I63.119, I63.12, I63.131, I63.132, I63.133, I63.139, I63.19, I63.20, I63.211, I63.212, I63.213, I63.219, I63.22, I63.231, I63.232,  I63.233, I63.239, I63.29, I63.30, I63.311, I63.312, I63.313, I63.319, I63.321, I63.322, I63.323, I63.329, I63.331, I63.332, I63.333, I63.339, I63.341, I63.342, I63.343, I63.349, I63.39, I63.40, I63.411, I63.412, I63.413, I63.419, I63.421, I63.422, I63.423, I63.429,  I63.431, I63.432, I63.433, I63.439, I63.441, I63.442, I63.443, I63.449, I63.49, I63.50, I63.511, I63.512, I63.513, I63.519, I63.521, I63.522, I63.523, I63.529, I63.531, I63.532, I63.533, I63.539, I63.541, I63.542, I63.543, I63.549, I63.59, I63.6, I63.8, I63.81, I63.89, I63.9, G43.601, G43.609, G43.611, G43.619 | -- |
| Anterior Cerebral Artery Ischemic Stroke | I63.321, I63.322, I63.323, I63.329, I63.421, I63.422, I63.423, I63.429, I63.521, I63.522, I63.523, I63.529 | -- |
| Basilar Artery Ischemic Stroke | I63.02, I63.12, I63.22 | -- |
| Carotid Artery Ischemic Stroke | I63.031, I63.032, I63.033, I63.039, I63.131, I63.132, I63.133, I63.139, I63.231, I63.232, I63.233, I63.239 | -- |
| Cerebellar Artery Ischemic Stroke | I63.341, I63.342, I63.343, I63.349, I63.441, I63.442, I63.443, I63.449, I63.541, I63.542, I63.543, I63.549 | -- |
| Middle Cerebral Artery Ischemic Stroke | I63.311, I63.312, I63.313, I63.319, I63.411, I63.412, I63.413, I63.419, I63.511, I63.512, I63.513, I63.519 | -- |
| Other Unspecified Artery Ischemic Stroke | G43.601, 643.609, G43.611, G43.619, I63.00, I63.09, I63.10, I63.19, I63.20, I63.29, I63.30, I63.39, I63.40, I63.49, I63.50, I63.59, I63.6, I63.8, I63.89, I63.9 | -- |
| Posterior Cerebral Artery Ischemic Stroke | I63.331, I63.332, I63.333, I63.339, I63.431, I63.432, I63.433, I63.439, I63.531, I63.532, I63.533, I63.539 | -- |
| Unspecified Small Artery Ischemic Stroke | I63.81 | -- |
| Vertebral Artery Ischemic Stroke | I63.011, I63.012, I63.013, I63.019, I63.111, I63.112, I63.113, I63.119, I63.211, I63.212, I63.213, I63.219 | -- |
| Anterior Circulation Acute Ischemic Stroke | I63.321, I63.322, I63.323, I63.329, I63.421, I63.422, I63.423, I63.429, I63.521, I63.522, I63.523, I63.529, I63.031, I63.032, I63.033, I63.039, I63.131, I63.132, I63.133, I63.139, I63.231, I63.232, I63.233, I63.239, I63.311, I63.312, I63.313, I63.319, I63.411, I63.412, I63.413, I63.419, I63.511, I63.512, I63.513, I63.519 | -- |
| Posterior Circulation Acute Ischemic Stroke | I63.02, I63.12, I63.22, I63.341, I63.342, I63.343, I63.349, I63.441, I63.442, I63.443, I63.449, I63.541, I63.542, I63.543, I63.549, I63.331, I63.332, I63.333, I63.339, I63.431, I63.432, I63.433, I63.439, I63.531, I63.532, I63.533, I63.539, I63.011, I63.012, I63.013, I63.019, I63.111, I63.112, I63.113, I63.119, I63.211, I63.212, I63.213, I63.219 | -- |
| Unspecified Circulation Acute Ischemic Stroke | G43.601, 643.609, G43.611, G43.619, I63.00, I63.09, I63.10, I63.19, I63.20, I63.29, I63.30, I63.39, I63.40, I63.49, I63.50, I63.59, I63.6, I63.8, I63.81, I63.89, I63.9 | -- |
| Embolic Acute Ischemic Stroke | Prefix: I63.0, I63.3, I63.6 | -- |
| Thrombotic Acute Ischemic Stroke | Prefix: I63.1, I63.4 | -- |
| Unspecified Etiology Acute Ischemic Stroke | Prefix: I63.2, I63.5, I63.8, I63.9, G43.6 | -- |
| National Institute of Health Stroke Scale (NIHSS) | R29.7xx (xx = 00-42) | -- |
| Endovascular Thrombectomy | 03CG3Z6, 03CG3Z7, 03CG3ZZ, 03CG4Z6, 03CG4ZZ, 03CH3Z6, 03CH3Z7, 03CH3ZZ, 03CH4Z6, 03CH4ZZ, 03CJ3Z6, 03CJ3Z7, 03CJ3ZZ, 03CJ4Z6, 03CJ4ZZ, 03CK3Z6, 03CK3Z7, 03CK3ZZ, 03CK4Z6, 03CK4ZZ, 03CL3Z6, 03CL3Z7, 03CL3ZZ, 03CL4Z6, 03CL4ZZ, 03CM3Z6, 03CM3Z7, 03CM3ZZ, 03CM4Z6, 03CM4ZZ, 03CN3Z6, 03CN3Z7, 03CN3ZZ,  03CN4Z6, 03CN4ZZ, 03CP3Z6, 03CP3Z7, 03CP3ZZ, 03CP4Z6, 03CP4ZZ, 03CQ3Z6, 03CQ3Z7, 03CQ3ZZ, 03CQ4Z6, 03CQ4ZZ | -- |
| Intravascular Thrombolysis | 3E03016, 3E03017, 3E03316, 3E03317, 3E04016, 3E04017, 3E04316, 3E04317,  3E05016, 3E05017, 3E05316, 3E05317, 3E06016, 3E06017, 3E06316, 3E06317,  3E07016, 3E07017, 3E07316, 3E07317, 3E08016, 3E08017, 3E08316, 3E08317,  Z92.82 | -- |
| Age | -- | AGE |
| Sex | -- | FEMALE |
| Race | -- | RACE |
| Income Quartile | -- | ZIPINC_QRTL |
| Expected Primary Payer | -- | PAY1 |
| Disposition of patient | -- | DISPUNIFORM |
| Length of Stay | -- | LOS |
| Region of Hospital | -- | HOSP_DIVISION |
| Patient Location: NCHS Urban-Rural Code | -- | PL_NCHS |
| Total Charges | -- | TOTCHG |
| APR-DRG Risk of Mortality | -- | APRDRG_Risk_Mortality |
| APR-DRG Severity of Illness | -- | APRDRG_Severity |
| NIS Hospital Number | -- | HOSP_NIS |
| Stratum used to post-stratify hospital | -- | NIS_STRATUM |
| Weight to discharges in the universe | -- | DISCWT |
| Admission Month | -- | AMONTH |
| Calendar Year | -- | YEAR |
