# Supplementary material for: Trends and disparities in NIHSS reporting and outcomes in acute ischemic stroke hospitalizations: A retrospective cross-sectional study
Source: Acta Neurochir (Wien). 2026 Apr 21;168(1):126. doi: 10.1007/s00701-026-06870-y (PMC13234067; doi:10.1007/s00701-026-06870-y)
Supplement: Supplementary file 2 — Supplementary file2 (DOCX 20 KB) [file 701_2026_6870_MOESM2_ESM.docx]

**Table S2.** Overall Monthly Reporting Rates of NIHSS Scores in AIS Hospitalizations

| **Year** | | **Month** | **Number of AIS Hospitalizations** | **Number of Hospitalizations with**  **Reported NIHSS (%)** |
| --- | --- | --- | --- | --- |
| 2016 | May | | 53590 | <11 |
| 2016 | June | | 51920 | <11 |
| 2016 | July | | 52510 | 0 (0) |
| 2016 | August | | 53750 | <11 |
| 2016 | September | | 51020 | 610 (1.20) |
| 2016 | October | | 53610 | 6715 (12.53) |
| 2016 | November | | 53195 | 8125 (15.27) |
| 2016 | December | | 55390 | 7935 (14.33) |
| 2017 | January | | 57440 | 10550 (18.37) |
| 2017 | February | | 51930 | 10250 (19.74) |
| 2017 | March | | 56670 | 11400 (20.12) |
| 2017 | April | | 55015 | 12265 (22.29) |
| 2017 | May | | 55940 | 12495 (22.34) |
| 2017 | June | | 53770 | 13040 (24.25) |
| 2017 | July | | 54525 | 13760 (25.24) |
| 2017 | August | | 55830 | 14530 (26.03) |
| 2017 | September | | 53020 | 14415 (27.19) |
| 2017 | October | | 56270 | 16115 (28.64) |
| 2017 | November | | 55300 | 15405 (27.86) |
| 2017 | December | | 56115 | 15355 (27.36) |
| 2018 | January | | 60455 | 18220 (30.14) |
| 2018 | February | | 51910 | 16050 (30.92) |
| 2018 | March | | 57680 | 18715 (32.45) |
| 2018 | April | | 56150 | 18540 (33.02) |
| 2018 | May | | 57725 | 21350 (36.99) |
| 2018 | June | | 55655 | 22135 (39.77) |
| 2018 | July | | 56190 | 24415 (43.45) |
| 2018 | August | | 56405 | 25180 (44.64) |
| 2018 | September | | 53960 | 24700 (45.77) |
| 2018 | October | | 57320 | 26130 (45.59) |
| 2018 | November | | 55045 | 25005 (45.43) |
| 2018 | December | | 56960 | 24585 (43.16) |
| 2019 | January | | 59670 | 27925 (46.80) |
| 2019 | February | | 53965 | 25855 (47.91) |
| 2019 | March | | 59590 | 28940 (48.57) |
| 2019 | April | | 57865 | 28400 (49.08) |
| 2019 | May | | 59920 | 29735 (49.62) |
| 2019 | June | | 57295 | 28890 (50.42) |
| 2019 | July | | 59740 | 30375 (50.85) |
| 2019 | August | | 58120 | 29435 (50.65) |
| 2019 | September | | 55940 | 28700 (51.30) |
| 2019 | October | | 59610 | 30030 (50.38) |
| 2019 | November | | 58355 | 29580 (50.69) |
| 2019 | December | | 59430 | 29730 (50.03) |
| 2020 | January | | 61885 | 31780 (51.35) |
| 2020 | February | | 57640 | 29440 (51.08) |
| 2020 | March | | 53105 | 27790 (52.33) |
| 2020 | April | | 46620 | 23690 (50.82) |
| 2020 | May | | 53870 | 27925 (51.84) |
| 2020 | June | | 55230 | 28895 (52.32) |
| 2020 | July | | 57515 | 30155 (52.43) |
| 2020 | August | | 56960 | 29750 (52.23) |
| 2020 | September | | 56475 | 30090 (53.28) |
| 2020 | October | | 58105 | 30495 (52.48) |
| 2020 | November | | 56875 | 29565 (51.98) |
| 2020 | December | | 59125 | 31205 (52.78) |
| 2021 | January | | 60685 | 30765 (50.70) |
| 2021 | February | | 54735 | 28420 (51.92) |
| 2021 | March | | 61025 | 32120 (52.63) |
| 2021 | April | | 59005 | 31070 (52.66) |
| 2021 | May | | 59640 | 32250 (54.07) |
| 2021 | June | | 60340 | 32375 (53.65) |
| 2021 | July | | 60100 | 32740 (54.48) |
| 2021 | August | | 59135 | 31915 (53.97) |
| 2021 | September | | 57200 | 31185 (54.52) |
| 2021 | October | | 59090 | 32275 (54.62) |
| 2021 | November | | 58630 | 32650 (55.69) |
| 2021 | December | | 60695 | 32375 (53.34) |
| 2022 | January | | 61650 | 33260 (53.95) |
| 2022 | February | | 54940 | 30215 (55.00) |
| 2022 | March | | 59900 | 34010 (56.78) |
| 2022 | April | | 58350 | 33365 (57.18) |
| 2022 | May | | 60440 | 34455 (57.01) |
| 2022 | June | | 58705 | 33775 (57.53) |
| 2022 | July | | 59400 | 34160 (57.51) |
| 2022 | August | | 59110 | 34135 (57.75) |
| 2022 | September | | 57490 | 33065 (57.51) |
| 2022 | October | | 59595 | 34165 (57.33) |
| 2022 | November | | 59865 | 34390 (57.45) |
| 2022 | December | | 60295 | 33925 (56.27) |
