# Supplementary material for: Trends and disparities in NIHSS reporting and outcomes in acute ischemic stroke hospitalizations: A retrospective cross-sectional study
Source: Acta Neurochir (Wien). 2026 Apr 21;168(1):126. doi: 10.1007/s00701-026-06870-y (PMC13234067; doi:10.1007/s00701-026-06870-y)
Supplement: Supplementary file 3 — Supplementary file3 (DOCX 15 KB) [file 701_2026_6870_MOESM3_ESM.docx]

**Table S3**. National Inpatient Sample Data Element Subgroups for Analysis

| **Variable** | **NIS Data Subgroups** |
| --- | --- |
| Age | <18, 18-39, 40-59, 60-79, ≥80 years |
| Sex | Male, Female |
| Race | White, Black, Hispanic, Asian or Pacific Islander, Native American, Other |
| Income Quartile | Q1, Q2, Q3, Q4 |
| Expected Primary Payer | Medicare, Medicaid, Private insurance, Self-pay, No charge, Other |
| Hospital Location | New England, Middle Atlantic, East North Central, West North Central, South Atlantic, East South Central, West South Central, Mountain, Pacific |
| Hospital Teaching Status | Rural, Urban Nonteaching, Urban Teaching |
| Hospital Bed Size | Small, Medium, Large |
| National Center for Health Statistics (NCHS) Urban-Rural Classification Scheme | "Central" counties of metro areas of >=1 million population, "Fringe" counties of metro areas of >=1 million population, Counties in metro areas of 250,000-999,999 population, Counties in metro areas of 50,000-249,999 population, Micropolitan counties, Not metropolitan or micropolitan counties |
| APR-DRG Risk of Mortality | No class specified, Minor likelihood of dying, Moderate likelihood of dying, Major likelihood of dying, Extreme likelihood of dying |
| Treatment Group | No reperfusion therapy, IVT alone, EVT alone, EVT and IVT |
| Discharge Disposition | Routine; Transfer to Short-term Hospital; Transfer Other: Includes Skilled Nursing Facility (SNF), Intermediate Care Facility (ICF), Another Type of Facility; Home Health Care (HHC); Against Medical Advice (AMA); Died; Discharge alive, destination unknown |
