# Supplementary material for: Trends and disparities in NIHSS reporting and outcomes in acute ischemic stroke hospitalizations: A retrospective cross-sectional study
Source: Acta Neurochir (Wien). 2026 Apr 21;168(1):126. doi: 10.1007/s00701-026-06870-y (PMC13234067; doi:10.1007/s00701-026-06870-y)
Supplement: Supplementary file 4 — Supplementary file4 (DOCX 16 KB) [file 701_2026_6870_MOESM4_ESM.docx]

**Table S4.** Stroke Subtype Classification Stratified by NIH Stroke Scale Documentation

|  |  | **NIHSS Documented (%)** | **NIHSS Not Documented (%)** | **P-Value** |
| --- | --- | --- | --- | --- |
|  |  | 1930880 | 2628029 |  |
| Infarcted Artery | Anterior Cerebral Artery | 29325 (1.5) | 33165 (1.3) | **<0.001** |
|  | Basilar Artery | 22360 (1.2) | 20420 (0.8) |  |
|  | Carotid Artery | 115605 (6.0) | 114170 (4.3) |  |
|  | Cerebellar Artery | 50955 (2.6) | 72080 (2.7) |  |
|  | Middle Cerebral Artery | 540340 (28.0) | 445530 (17.0) |  |
|  | Other Unspecified Artery | 913180 (47.3) | 1703924 (64.8) |  |
|  | Posterior Cerebral Artery | 90575 (4.7) | 95600 (3.6) |  |
|  | Unspecified Small Artery | 137015 (7.1) | 112200 (4.3) |  |
|  | Vertebral Artery | 31525 (1.6) | 30940 (1.2) |  |
|  |  |  |  |  |
| Stroke Etiology | Embolic | 413955 (21.4) | 478660 (18.2) | **<0.001** |
|  | Thrombotic | 124175 (6.4) | 106440 (4.1) |  |
|  | Unspecified | 1392750 (72.1) | 2042929 (77.7) |  |
|  |  |  |  |  |
| Infarct Location | Anterior Circulation | 689305 (35.7) | 596615 (22.7) | **<0.001** |
|  | Posterior Circulation | 193775 (10.0) | 218830 (8.3) |  |
|  | Unspecified Location | 1047800 (54.3) | 1812584 (69.0) |  |
