# Supplementary material for: Trends and disparities in NIHSS reporting and outcomes in acute ischemic stroke hospitalizations: A retrospective cross-sectional study
Source: Acta Neurochir (Wien). 2026 Apr 21;168(1):126. doi: 10.1007/s00701-026-06870-y (PMC13234067; doi:10.1007/s00701-026-06870-y)
Supplement: Supplementary file 5 — Supplementary file5 (DOCX 20 KB) [file 701_2026_6870_MOESM5_ESM.docx]

**Table S5.** Monthly Reporting Rates of NIHSS Scores in Thrombectomy Centers

| **Year** | **Month** | **Total Number of AIS Hospitalizations** | **AIS Hospitalizations with**  **Reported NIHSS Scores (%)** |
| --- | --- | --- | --- |
| 2016 | May | 21770 | <11 |
| 2016 | June | 21315 | <11 |
| 2016 | July | 21815 | 0 (0) |
| 2016 | August | 22270 | <11 |
| 2016 | September | 20520 | 320 (1.56) |
| 2016 | October | 21710 | 3980 (18.33) |
| 2016 | November | 21940 | 4750 (21.65) |
| 2016 | December | 23010 | 4750 (20.64) |
| 2017 | January | 25910 | 6755 (26.07) |
| 2017 | February | 23805 | 6450 (27.10) |
| 2017 | March | 26360 | 7420 (28.15) |
| 2017 | April | 25460 | 7810 (30.68) |
| 2017 | May | 25885 | 7790 (30.09) |
| 2017 | June | 25255 | 8165 (32.33) |
| 2017 | July | 25285 | 8655 (34.23) |
| 2017 | August | 25925 | 8895 (34.31) |
| 2017 | September | 24485 | 8945 (36.53) |
| 2017 | October | 26490 | 9980 (37.67) |
| 2017 | November | 25170 | 9070 (36.03) |
| 2017 | December | 25955 | 9315 (35.89) |
| 2018 | January | 30295 | 11810 (38.98) |
| 2018 | February | 26340 | 10450 (39.67) |
| 2018 | March | 29185 | 12065 (41.34) |
| 2018 | April | 28515 | 11795 (41.36) |
| 2018 | May | 29645 | 13735 (46.33) |
| 2018 | June | 28695 | 14100 (49.14) |
| 2018 | July | 29295 | 15345 (52.38) |
| 2018 | August | 28880 | 15525 (53.76) |
| 2018 | September | 27865 | 15545 (55.79) |
| 2018 | October | 29560 | 16205 (54.82) |
| 2018 | November | 28540 | 15610 (54.70) |
| 2018 | December | 29250 | 15270 (52.21) |
| 2019 | January | 31005 | 17105 (55.17) |
| 2019 | February | 28265 | 15845 (56.06) |
| 2019 | March | 31200 | 17750 (56.89) |
| 2019 | April | 30285 | 17365 (57.34) |
| 2019 | May | 31710 | 18255 (57.57) |
| 2019 | June | 30390 | 17935 (59.02) |
| 2019 | July | 31585 | 18520 (58.64) |
| 2019 | August | 31015 | 18065 (58.25) |
| 2019 | September | 29790 | 17325 (58.16) |
| 2019 | October | 31810 | 18225 (57.29) |
| 2019 | November | 30935 | 18065 (58.40) |
| 2019 | December | 31310 | 18180 (58.06) |
| 2020 | January | 32905 | 19425 (59.03) |
| 2020 | February | 29780 | 17390 (58.39) |
| 2020 | March | 28030 | 16605 (59.24) |
| 2020 | April | 24395 | 14190 (58.17) |
| 2020 | May | 28395 | 16660 (58.67) |
| 2020 | June | 29040 | 17355 (59.76) |
| 2020 | July | 30375 | 18425 (60.66) |
| 2020 | August | 30265 | 17945 (59.29) |
| 2020 | September | 29725 | 17840 (60.02) |
| 2020 | October | 30625 | 18200 (59.43) |
| 2020 | November | 29840 | 17650 (59.15) |
| 2020 | December | 31115 | 18630 (59.87) |
| 2021 | January | 32725 | 19080 (58.30) |
| 2021 | February | 29640 | 17475 (58.96) |
| 2021 | March | 32915 | 19440 (59.06) |
| 2021 | April | 31955 | 18975 (59.38) |
| 2021 | May | 32545 | 19805 (60.85) |
| 2021 | June | 32420 | 19565 (60.35) |
| 2021 | July | 32200 | 19885 (61.75) |
| 2021 | August | 31810 | 19615 (61.66) |
| 2021 | September | 30385 | 18890 (62.17) |
| 2021 | October | 31390 | 19465 (62.01) |
| 2021 | November | 31675 | 19940 (62.95) |
| 2021 | December | 32430 | 19345 (59.65) |
| 2022 | January | 33695 | 20230 (60.04) |
| 2022 | February | 29965 | 18375 (61.32) |
| 2022 | March | 32980 | 20550 (62.31) |
| 2022 | April | 32290 | 20510 (63.52) |
| 2022 | May | 33570 | 21110 (62.88) |
| 2022 | June | 32560 | 20620 (63.33) |
| 2022 | July | 33445 | 21040 (62.91) |
| 2022 | August | 32465 | 20470 (63.05) |
| 2022 | September | 31730 | 19870 (62.62) |
| 2022 | October | 32960 | 20605 (62.52) |
| 2022 | November | 33210 | 20970 (63.14) |
| 2022 | December | 33350 | 20605 (61.78) |
