# Supplementary figures and images for: Trends and disparities in NIHSS reporting and outcomes in acute ischemic stroke hospitalizations: A retrospective cross-sectional study
Source: Acta Neurochir (Wien). 2026 Apr 21;168(1):126. doi: 10.1007/s00701-026-06870-y (PMC13234067; doi:10.1007/s00701-026-06870-y)

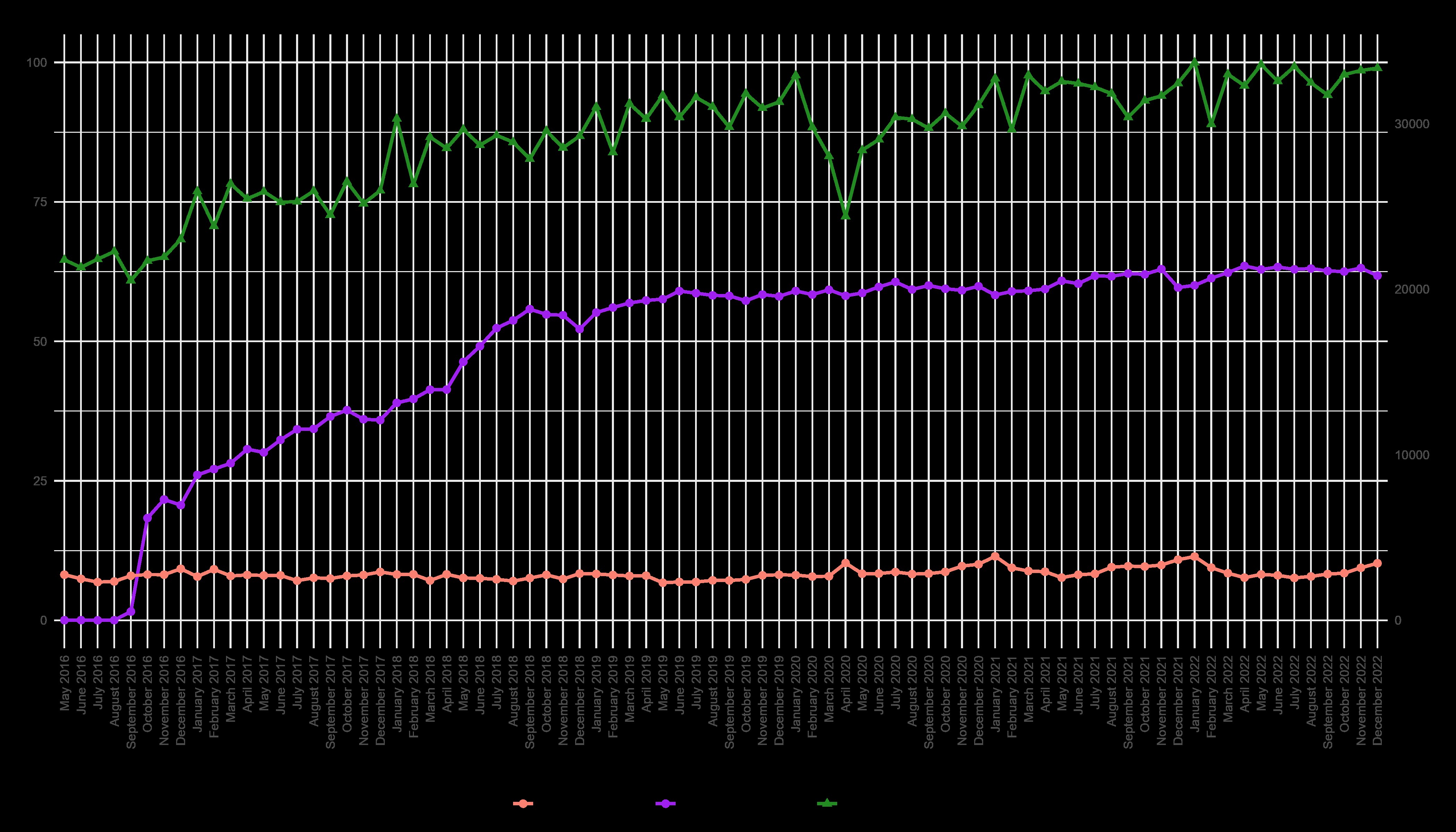

Supplement: Supplementary file 6 — Supplementary file6 (JPEG 1258 KB) [file 701_2026_6870_MOESM6_ESM.jpeg]

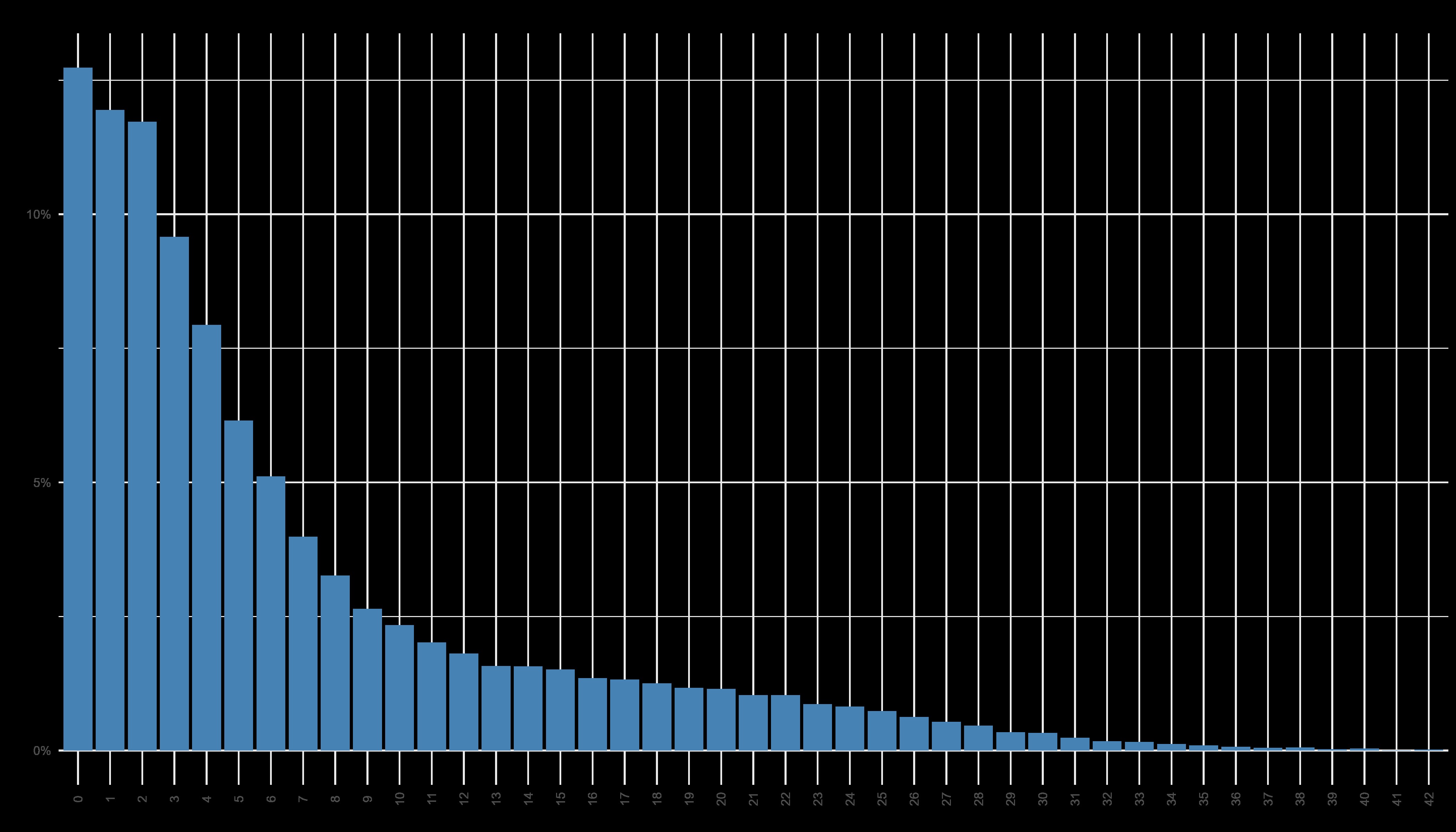

Supplement: Supplementary file 7 — Supplementary file7 (JPEG 624 KB) [file 701_2026_6870_MOESM7_ESM.jpeg]
